# Screening of the college students at clinical high risk for psychosis in China: a multicenter epidemiological study

**DOI:** 10.1186/s12888-021-03229-8

**Published:** 2021-05-17

**Authors:** Jiaxin Wu, Xiangyun Long, Fei Liu, Ansi Qi, Qi Chen, Xiaofeng Guan, Qiong Zhang, Yuhong Yao, Jingyu Shi, Shiping Xie, Wei Yan, Maorong Hu, Xin Yuan, Jun Tang, Siliang Wu, Tianhong Zhang, Jijun Wang, Zheng Lu

**Affiliations:** 1 Department of Psychiatry, Tongji Hospital of Tongji University, Tongji University School of Medicine, 389 Xincun Road, Shanghai, 200065 PR China; 2 Tongji University School of Medicine, Shanghai, 200092 PR China; 3 Department of Psychiatry, Affiliated Nanjing Brain Hospital, Nanjing Medical University, Nanjing, 210029 PR China; 4 Department of Psychiatry, The First Affiliated Hospital of Nanchang University, Nanchang, 330006 PR China; 5 Shanghai Mental Health Center, Shanghai Jiaotong University School of Medicine, Shanghai Key Laboratory of Psychotic Disorders, Shanghai, 200030 PR China

**Keywords:** Clinical high risk, Ultra-high risk, Early detection, College students, Epidemiology

## Abstract

**Background:**

To investigate a 3-stage screening procedure and explore the clinical features of subjects at Clinical High Risk (CHR) for psychosis in a representative sample of Chinese college students.

**Methods:**

An epidemiological survey of the prevalence of the CHR syndrome in Chinese college students that was selected by stratified random sampling from Shanghai, Nanjing and Nanchang cities was done following a 3-stage procedure. Participants were initially screened with the Prodromal Questionnaire-brief version (PQ-B), and whose distress score of PQ-B exceeded 24 would be invited to a telephone assessment using the subscale for positive symptoms of the Scale of Prodromal Symptoms (SOPS)/Structured Interview for Prodromal Syndromes (SIPS). Lastly, participants who scored 3 or higher in any item of the subscale would be administered with the SIPS interview conducted by trained researchers to confirm the diagnosis of CHR syndrome.

**Results:**

Twenty-three thousand sixty-three college students completed the survey during September 2017 to October 2018. Seventy-two students were diagnosed as CHR subjects, and the detection rate in the total sample was 0.3%. The peak age range for the first diagnosis of CHR was 17 to 20 years. Thirteen and forty-six were set as the cutoff points of PQ-B total score and distress score to balance the greatest sensitivity and specificity. Binary logistic regression revealed that 8 items in PQ-B showed significant distinction for detecting CHR subjects.

**Conclusions:**

The 3-stage screening method can be utilized in the detection of CHR subjects for psychosis in the general population, during which delusional ideas, perceptual abnormalities and suspiciousness deserve great attention.

**Supplementary Information:**

The online version contains supplementary material available at 10.1186/s12888-021-03229-8.

## Background

Since being proposed by Sullivan in 1927, the notion of early detection and preventive intervention of psychosis syndromes has received increased attention in the recent two decades [1–4]. As a series of studies of first-episode psychosis suggests that longer duration of untreated psychosis significantly correlates with worse treatment response and prognosis [5], global researchers and clinicians have reached a consensus that early detection and prevention is central to contain the development and burden of psychosis. They started to investigate whether there is an earlier period in the development of psychosis that can be detected before the break, and then gave birth to the concept of ultra-high risk (UHR) or clinical high risk (CHR) for psychosis [6, 7]. CHR subjects refer to individuals who are identified as having nonspecific symptoms (e.g., cognitive symptoms) and attenuated or intermittent psychotic symptoms, schizotypal personality traits or having asymptomatic genetic risks, but do not meet criteria for a syndromal psychosis, and thereby they are considered to be at increased risk for developing psychotic disorders [4, 8]. Fusar-Poli et al. [9] reported that 20–35% CHR subjects developed syndromal psychotic disorder in 2 years through a meta-analysis, and Zhang et al. [10] discovered that the transition rate in the first 2 years was 26.4% in a Chinese help-seeking sample, both of which indicated that the risk of developing psychosis in CHR subjects deserved serious attention.

To diagnose and assess symptoms of CHR, two categories of instruments based on two complementary criteria have been globally recognized: the UHR criteria that focus on attenuated or brief limited intermittent psychotic symptoms and genetic risk, and the basic symptoms criteria that focus on cognitive and perceptive symptoms. The UHR criteria were designed to detect the risk for developing a first-episode psychosis in 12 months [11]. The most recognized instruments developed from the UHR criteria include the Structured Interview for Psychosis-risk Syndromes (SIPS) [12] and the Comprehensive Assessment of At-Risk Mental States (CAARMS) [13]. While the basic symptoms criteria aimed at detecting the risk for psychosis ideally before the appearance of functional impairments [11]. The most representative instruments are the Schizophrenia Proneness Instrument, Adult (SPI-A) [14] and Child & Youth version (SPI-CY) [15]. Since the instruments listed above are based on the clinician’s assessments through clinical interviews, several self-report scales have been established to simplify the screening of CHR individuals, such as Prodromal Questionnaire-Brief version (PQ-B) [16], Prodromal Questionnaire-16 items (PQ-16) [17], Prime Screen [18] and Early Psychosis Screener (EPS) [19, 20].

Owing to the development of the diagnostic instruments, the screening of CHR have been conducted globally, especially in samples of clinician-referred patients and help-seeking individuals. However, since some of the CHR subjects won’t see a doctor (e.g. because of social discrimination, stigma or poor knowledge of psychosis) before the onset of psychosis or significant functional impairments [21], screening CHR in clinical samples appeared to be a passive method. Therefore, detecting CHR in general populations have raised more and more concerns recently. Besides, as the risk age range of CHR is 15 to 22 years, great attention was paid to the studies carried out in students in universities and high schools [22]. Notwithstanding, detection of CHR in China is still limited by its passivity that the clinical interviews and rating scales can almost only be approached in psychiatric clinics or wards, which indicates that only individuals with help-seeking behaviors can be tested, and doctors should always wait passively for the suspected cases. In fact, most researches concerning CHR detection in China were conducted in help-seeking individuals [10, 23, 24]. Only few studies attempted to detect CHR in Chinese general populations. For instance, Chen et al. established a two-stage screening method (PQ-16 followed by SIPS) in 579 Chinese university students who were enrolled in a mental health education course, and reported that the prevalence of CHR was 1.1% [25]. However, the prevalence rates of CHR varied widely in the literatures, ranging from 0.3 to 2.4% [26–28]. Therefore, an epidemiological study of CHR detection conducted in a larger sample of Chinese generalpopulations with established psychosis-risk instruments will be of great significance. In this study, we generalized a 3-stage method (consists of PQ-B, telephone interview and SIPS) for the detection of CHR individuals in college students from Shanghai, Nanjing and Nanchang in China. The present study aimed to investigate the screening procedure, and explore the characteristics of prodromal symptoms of psychosis in Chinese college students. To our knowledge, this is the first multicentre epidemiological study on the detection of CHR subjects in Chinese college students.

## Method

### Participants

The Research Ethics Committee at the Shanghai Mental Health Centre (SMHC), Shanghai, China approved this study in 2016. Participants were college students recruited by three research teams in three different cities: Tongji Hospital of Tongji University (TJH) at Shanghai, Nanjing Brain Hospital (NBH) at Nanjing, and The First Affiliated Hospital of Nanchang University (FAHNU) at Nanchang, from September 2017 to October 2018. Stratified random sampling was conducted at the school level, including the Tier-1 universities, Tier-2 universities, Tier-3 universities and higher vocational and technical colleges. The paper and online versions of the questionnaires were distributed to the selected sample of 27,145 students. Given that the written informed consent was not applicable in the online survey and telephone assessment, it was only obtained from the participants who received a paper questionnaire and a face-to-face interview. Nonetheless, online informed consent was provided as part of the online survey. Similarly, the informed consent was given verbally during the telephone assessment. Eventually, 26,005 questionnaires were received, and 2942 questionnaires were removed due to missing data including demographic and blank data in PQ-B items. Thus, a sample of 23,063 college students were investigated in this study. The age range of the investigated sample was 14 to 31 years (mean = 18.4 years, S.D. = 1.2 years), and 11,028 (47.8%) subjects were female.

### Instruments

#### The prodromal questionnaire-brief version (PQ-B)

The PQ-B [16] is a 21-item self-report scale designed to detect individuals at risk for psychosis. It was modified from the 92-item Prodromal Questionnaire [29] and focus on positive symptoms. Participants indicated “Yes/No” to each item depending on the presence or absence of the corresponding experience in the last month. If an item was marked as “No”, the item was rated as 0; if an item was marked as “Yes”, a 5-point Likert scale (strongly disagree, disagree, neutral, agree, strongly agree) was used to rate the distress level of the symptom. The total score was defined as the number of the items endorsed, and the distress score was the sum of the distress level of the items endorsed.

Zhang et al. [10] and Xu et al. [30] translated the original version of the PQ-B into simplified Mandarin Chinese, and reported good reliability and validity in a sample of help-seeking outpatients in China (Cronbach’s alpha = 0.897). They set the cutoff point for the total score and distress score of the Chinese-version PQ-B as 7 and 24 respectively. The sensitivity and specificity were reported to be 82.0 and 46.8% respectively when using the cutoff point for the distress score to screen CHR subjects. In the present study, the internal consistency of the Chinese-version PQ-B was excellent (Cronbach’s alpha = 0.928).

#### The structured interview for psychosis-risk syndromes/scale of prodromal syndromes (SIPS/SOPS)

The SIPS/SOPS [12] were designed to determine if an individual met the CHR criteria through a semi-structured clinical interview administered by a clinician. The criteria outlined three types of Psychosis-Risk Syndromes: Attenuated Positive Symptom Syndrome (APSS), Brief Intermittent Psychotic Syndrome (BIPS), and Genetic Risk and Deterioration Syndrome (GRDS). There are 19 items assessing 4 major symptom domains in the SIPS/SOPS, including Positive symptoms, Negative symptoms, Disorganized symptoms, and General Symptoms. Each item was rated from 1 to 6, according to the severity of the symptom. A rating of 3 to 5 indicates a prodromal range symptom, and 6 indicates a “severe and psychotic” symptom. Li et al. established the Chinese version of SIPS/SOPS, and it was demonstrated with good reliability and validity in assessing subjects with Psychosis-Risk Syndromes [31]. The internal consistency reliability of SIPS/SOPS in this study was good (Cronbach’s alpha = 0.848).

#### Demographic characteristics

Demographic characteristics that include name, sex, age, discipline, family history and contact information were measured with a questionnaire (see the Supplementary material) designed by the authors.

### Procedure

**Fig. 1:**
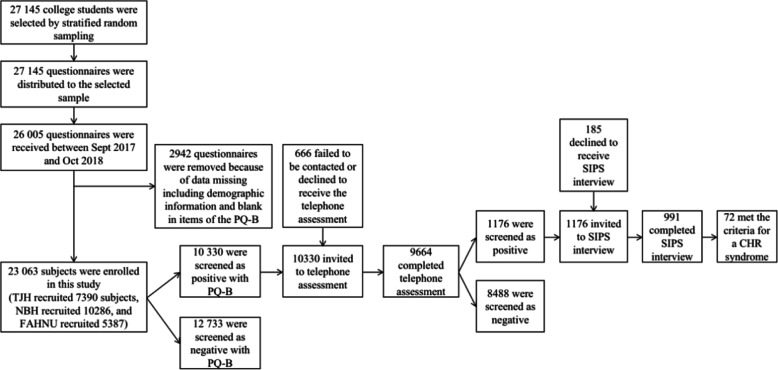
The sample flowchart of this study. TJH, Tongji Hospital of Tongji University; NBH, Nanjing Brain Hospital; FAHNU, The First Affiliated Hospital of Nanchang University

A 3-stage screening procedure (see Fig. 1) was applied in this study. The screening results for the Chinese-version of the PQ-B were obtained from 23,063 participants (the investigated sample). According to Xu et al. [30], the cutoff point of the distress score of PQ-B was set as 24 in this study. 10,330 (44.8%) participants reached the threshold, and were defined as the PQ-B-positive subjects, and were invited to a telephone assessment. Since 666 participants were failed to be contacted (e.g., dead number, suspended service or didn’t answer for three times) or declined to complete the telephone assessment. 9664 (41.9%) participants were assessed by trained researchers, with the items listed in the subscale for positive symptoms in the SIPS/SOPS (P1-P5, Unusual Thought Content, Suspiciousness/Persecutory Ideas, Grandiosity, Perceptual Abnormalities/Hallucinations, and Disorganized Communication) to identify the possible presence of the prodromal or psychotic symptoms. If any item was rated as 3 or higher, the participant would be considered as positive in the telephone assessment. Thus, 1176 participants were invited to the face-to-face interviews, and 991 (4.3%) participants eventually consented to receive the SIPS interviews administered by trained researchers. Participants who met any criterion of the Psychosis-Risk Syndromes (APSS, BIPS, GRDS) defined by SIPS were diagnosed as CHR individuals, while all other members of the investigated sample were classified into non-CHR individuals. All interviewers had received a professional training for SIPS prior to the study. The inter-rater reliability was good among the interviewers (*n* = 20, ICC = 0.75).

### Statistical analyses

The sample size of this study was estimated by the prevalence (p) of CHR syndrome, which was reported to be 0.3% in a young adults’ community [27]. Using the formula [32]:

$$ \mathrm{n}={\mathrm{Z}}_{1-\upalpha}^2\left(1-\mathrm{p}\right)/\mathrm{p}\upvarepsilon $$

with the relative error (ε) set at 25%, the sample size was calculated to be roughly 20,500. Considering a response rate of 80%, we were required to survey 25,625 individuals.

Using SPSS software (IBM Corp. Released 2017. IBM SPSS Statistics for Macintosh, Version 25.0. Armonk, NY: IBM Corp.), the group differences in age, the total score and distress score as well as each item score of the PQ-B were compared using Independent-samples t-tests, and the significance level was set at 0.05 (two-tailed). Likewise, Chi-square tests were used to compare sex between the two groups. Mann-Whitney U test was used to test whether there was a difference in disciplines between the two groups. As 60.8% of the data of the family history was missing, family history was not described in the demographic characteristics. The internal consistency reliability of PQ-B and SOPS were described using the Cronbach’s alpha. The receiver operating characteristic (ROC) analyses were performed to reset the cutoff points and to examine the sensitivity and specificity of PQ-B in detecting CHR subjects. The accuracy of the tests was described through the areas under the ROC curve (AUCs) with the 95% confidential interval (CI). A Spearman correlation analysis was used to investigate the association between each item score of PQ-B and the dichotomized SIPS diagnosis. A logistic regression analysis was conducted to explore key items of the PQ-B in the indication of CHR status based on the SIPS. For graphical presentation, bar diagrams were created to show the number of the CHR subjects by the age, as well as its distribution in female and male groups, and a bar-chart (mean and standard error of the mean) was created to show the differences in the PQ-B item scores between CHR and non-CHR subjects.

## Results

### Demographic characteristics

72 college students met the criteria for CHR according to the SIPS. The detection rate in the total sample was 0.3% (72/23063). Of those, 1 met the GRDS criteria, 71 met the APSS criteria, and none met the BIPS criteria. Demographic characteristics for CHR individuals and non-CHR individuals were presented in Table 1. No significant difference in the mean age, sex, and disciplines was found between the two groups.

**Table 1:** Demographic characteristics of the CHR and non-CHR individuals

Variables	CHR (*n* = 72)	Non-CHR (*n* = 22,991)	t / χ^2^/ U	p
Age mean (S.D.)	18.3 (1.5)	18.4 (1.2)	0.38	0.707
Female (%)	38 (52.8)	10,990 (47.8)	0.71	0.399
Disciplines				
Arts (%)	19 (26.4)	2502 (10.9)	751,769.50	0.243
Humanities (%)	4 (5.6)	1100 (4.8)	751,769.50	0.243
Social sciences (%)	3 (4.2)	2482 (10.8)	751,769.50	0.243
Natural sciences (%)	3 (4.2)	3355 (14.6)	751,769.50	0.243
Applied sciences (%)	40 (55.6)	12,769 (55.5)	751,769.50	0.243
Unclassified (%)	2 (2.8)	623 (2.7)	751,769.50	0.243
Null (%)	1 (1.4)	160 (0.7)	751,769.50	0.243
PQ-B total score mean (S.D.)	18.0 (4.4)	8.5 (7.1)	18.22	< 0.001
PQ-B distress score mean (S.D.)	66.0 (22.1)	24.6 (21.1)	16.64	< 0.001

### Age of CHR subjects at identification

**Fig. 2:**
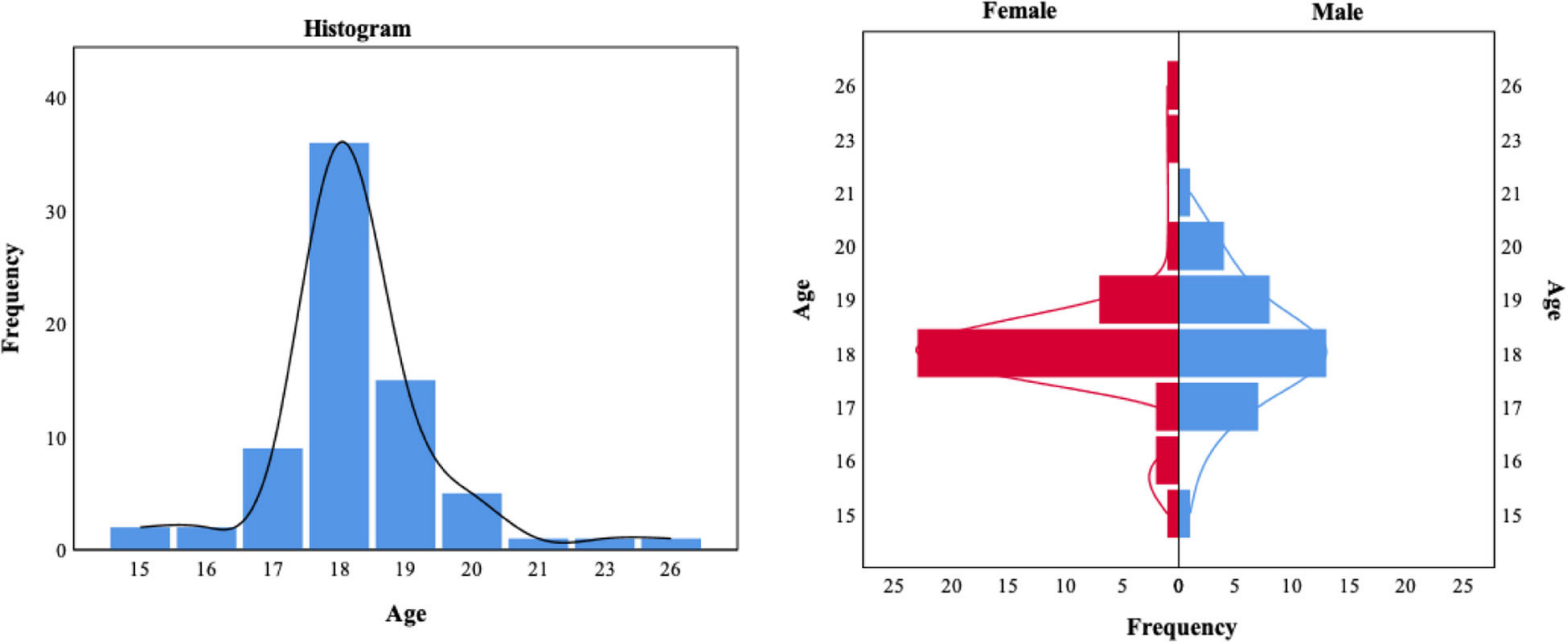
Age distribution for the total sample, females and males meeting CHR criteria

The age distribution of CHR subjects was shown in Fig. 2. The frequency of CHR identified with SIPS in this sample peaked at young adulthood (17 to 20 years) and decreased with subsequent age. The peak age that met the CHR criteria for both males and females was 18 years. Whereas the age range of female subjects (15 to 26 years) was broader than that of male subjects (15 to 21 years).

### Evaluative value of PQ-B in assessing CHR individuals

#### The total score and distress score of PQ-B in detecting CHR

**Fig. 3:**
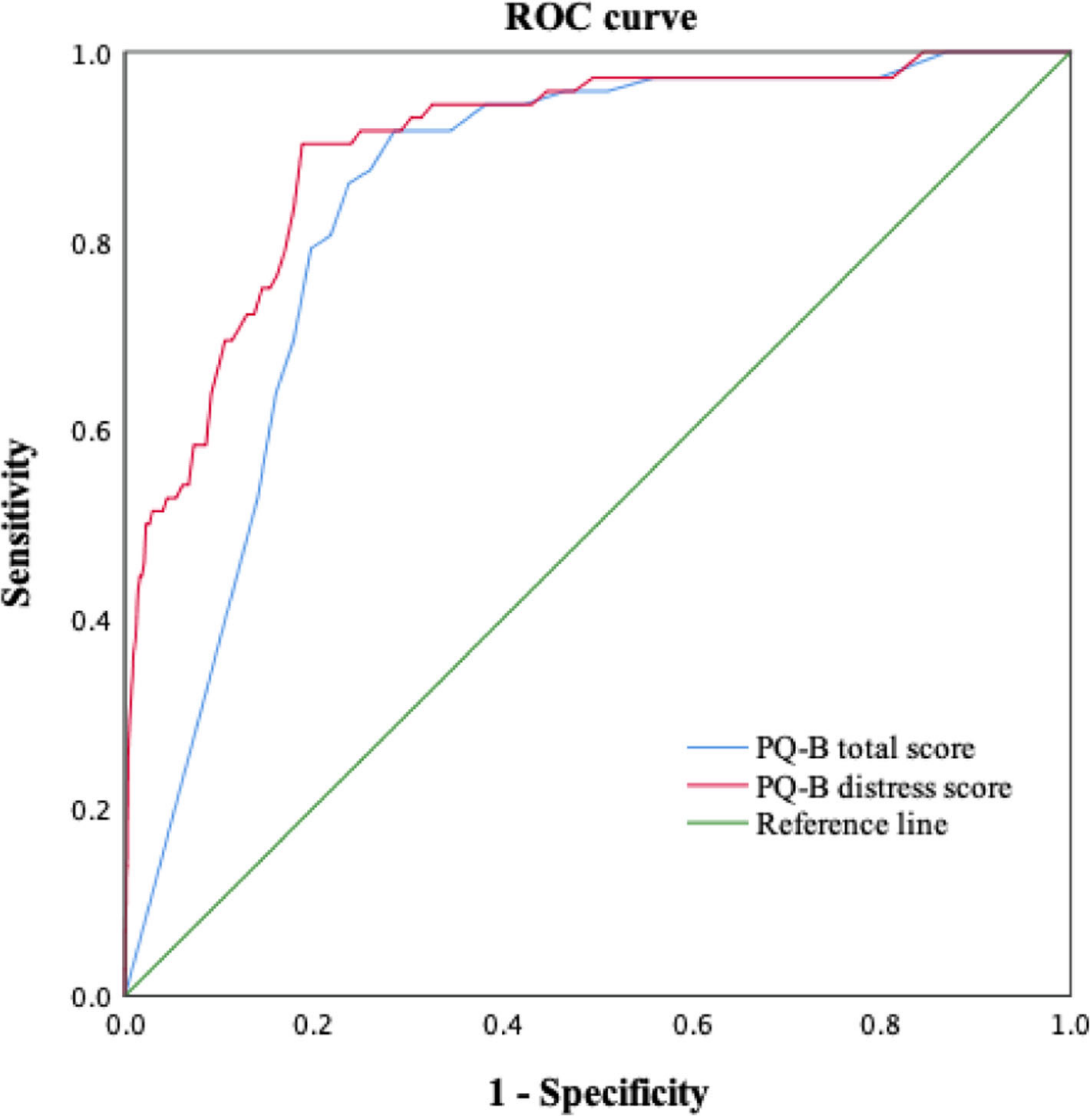
ROC curves for the total score and distress score of PQ-B as indicator of CHR diagnosis

Compared to non-CHR individuals, both the total and distress scores of PQ-B were significantly higher among CHR individuals (see Table 1). To examine the accuracy and to reset the cutoff points for Chinese college students, the diagnosis based on SIPS was set as the state variable, and the ROC analyses were performed for the PQ-B total score and distress score, whose AUCs were also calculated (see Fig. 3). Both the total score and distress score yielded significant value in assessing CHR individuals. The total score of PQ-B yielded an AUC of 0.842 (95% CI: 0.806, 0.877) for screening CHR, and the AUC of the distress score was 0.900 (95% CI: 0.863, 0.937). At different cutoff points for the PQ-B total score and distress score, the sensitivity and specificity values were calculated and presented in Table 2. The cutoff point of 13 for the total score, and 46 for the distress score balanced the greatest sensitivity and greatest specificity. For the total score, a cutoff point of 13 or higher possessed a sensitivity of 91.7%, and a specificity of 71.5%. While the sensitivity for the distress score when setting the cutoff point at 46 or higher was 90.3%, the corresponding specificity reached 81.2%.

**Table 2:** Accuracy of PQ-B total score and distress score in screening CHR

PQ-B cutoff	Sensitivity (%)	Specificity (%)	YI^a^	LR + ^a^	AUC^a^	95% CI	p
Total score							
≥ 10	94.4	61.9	0.56	2.48	0.842	0.806–0.877	< 0.001
≥ 11	91.7	65.6	0.57	2.67	0.842	0.806–0.877	< 0.001
≥ 12	91.7	68.9	0.61	2.95	0.842	0.806–0.877	< 0.001
≥ 13^b^	91.7	71.5	0.63	3.22	0.842	0.806–0.877	< 0.001
≥ 14	87.5	74.1	0.62	3.38	0.842	0.806–0.877	< 0.001
≥ 15	86.1	76.3	0.62	3.63	0.842	0.806–0.877	< 0.001
Distress score							
≥ 43	90.3	78.2	0.69	4.14	0.900	0.863–0.937	< 0.001
≥ 44	90.3	79.2	0.70	4.34	0.900	0.863–0.937	< 0.001
≥ 45	90.3	80.3	0.71	4.58	0.900	0.863–0.937	< 0.001
≥ 46^b^	90.3	81.2	0.72	4.80	0.900	0.863–0.937	< 0.001
≥ 47	83.3	82.2	0.66	4.68	0.900	0.863–0.937	< 0.001
≥ 48	79.2	83.0	0.62	4.66	0.900	0.863–0.937	< 0.001

^a^*YI* Youden’s index, *LR+* positive likelihood ratio, *AUC* area under the curve

^b^cutoff point with the highest YI

#### The significant items of PQ-B in detecting CHR

**Fig. 4:**
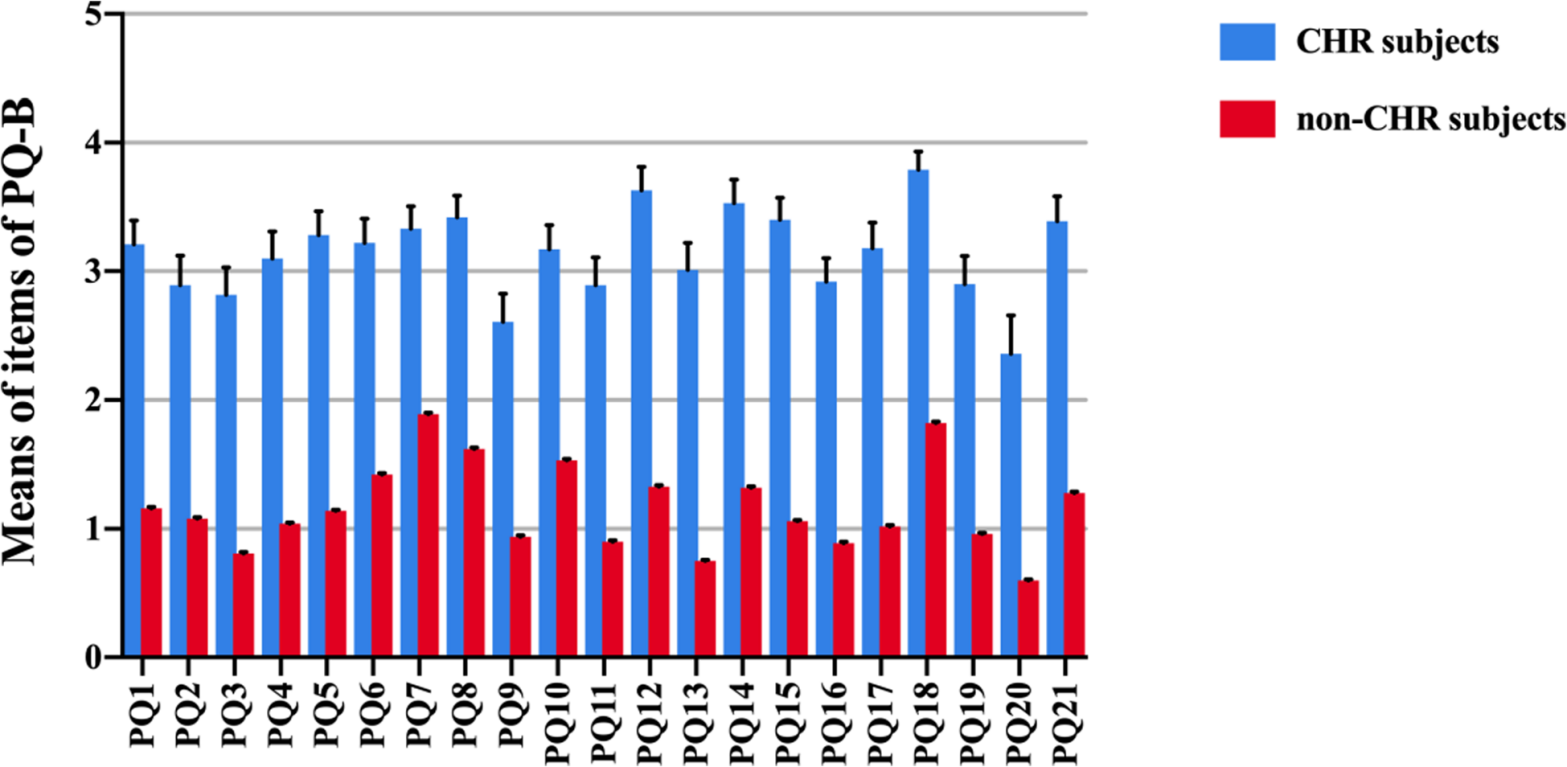
Comparison of PQ-B item scores in CHR and non-CHR individuals: the means of PQ-B items are significantly higher in CHR individuals (*p* < 0.001)

To investigate which items were significant in detecting CHR in Chinese college students, independent-samples t-tests were initially performed to compare the differences in the item scores of PQ-B between CHR and non-CHR individuals. The results demonstrated that the score for each individual item in PQ-B was significantly higher in the CHR group (all *p* < 0.001), which was presented in a bar chart (see Fig. 4). Additionally, a Spearman correlation analysis showed that higher score of each item in the PQ-B was significantly associated with the diagnosis of CHR (all p < 0.001). Therefore, we did a conditional backward logistic regression analysis to explore the key items involved in the detection of CHR subjects. All PQ-B item scores were set as the independent variables and the diagnosis (CHR or non-CHR) was set as the dependent variable. As presented in Table 3, 8 items (PQ1, PQ3, PQ4, PQ12, PQ13, PQ15, PQ17 and PQ18) were entered into the regression model.

**Table 3:** Logistic regression of the CHR/non-CHR classification by PQ-B items

Variables	B	S.E.	Wald	Df	p	Exp(B)
PQ1	0.223	0.086	6.764	1	0.009	1.249
PQ4	0.225	0.077	8.489	1	0.004	1.252
PQ12	0.240	0.094	6.504	1	0.011	1.271
PQ13	0.204	0.082	6.138	1	0.013	1.226
PQ15	0.282	0.103	7.527	1	0.006	1.326
PQ17	0.182	0.088	4.317	1	0.038	1.200
PQ18	0.199	0.064	9.666	1	0.002	1.220
PQ21	0.188	0.094	3.995	1	0.046	1.206
Constant	−9.813	0.468	439.389	1	< 0.001	0.000
Overall model fit test	χ^2^ = 252.494, p < 0.001; Hosmer–Lemeshow test value = 8.309, *p* = 0.306.					
Associated strength	Cox-Snell R^2^ = 0.011; Nagelkerke R^2^ = 0.263					

## Discussion

The detection of CHR samples in both the clinical settings [10, 33–36] and non-clinical settings [26–28] attracted a great deal of attention around the world, while the detection rate of CHR subjects in these studies varied considerably. Previous studies reported that the prevalence of CHR in the clinician-referred samples was 32–42% [33–35], which correspond roughly to one another. While the detection rate in samples of help-seeking individuals ranged from 4.2 to 80%, which varied from site to site [10, 37–39]. For the general population (primarily focused on young adults and adolescents), the annual incidence of new cases of CHR was estimated at 1/10000 [33], and the prevalence ranged from 0.3 to 2.4% [26–28]. The diagnostic instruments were considered to be a great factor contributing to the inconsistency of the detection rate. Schultze et al. [27] screened CHR in a young adult community with SIPS and SPI-A, and reported that 2.4% of the participants met at least one CHR criterion, while only 0.6% met the APS criteria identified by the SIPS. Salazar et al. [40] reviewed 56 studies and suggested that the 0.3% of the general non-help-seeking population of youth met the DSM-5-APS criteria, while 1.3% of the general population met the SIPS-APSS criteria.

In this study, the detection rate of CHR subjects identified based on the SIPS criteria in Chinese college students was 0.3%. Although it was lower than the detection rate (1.1%) reported in another study conducted in the Chinese college students [28], a multicentre sample and a random sampling method both proved the reliability of the results in a degree. Nevertheless, as is mentioned before, only about 30% of the CHR would make the transition to psychosis, and thus the prevalence of CHR should exceed that of psychotic disorders intheory. While in fact, the prevalence of schizophrenia and other psychotic disorders was about 0.7% [32], much higher than the detection rate of CHR reported in this study. A possible explanation for the contradiction may originate from the education level of the sample. Since only college students were recruited in this study, a large proportion of the CHR individuals who failed or even quitted the College Entrance Exam because of the disturbance of prodromal symptoms were not investigated, and thus it may be underestimated if regarding 0.3% as the prevalence of CHR in the Chinese general population.

Since first-episode psychosis usually occurs in late adolescence and early adulthood, CHR or prodromal psychosis were believed to have an earlier onset [41, 42]. Our research demonstrated that the peak age range of the CHR identification by SIPS was between 17 to 20 years in the sample of Chinese college students, which was largely in line with previous studies, but somewhat narrower (e.g., SHARP reported 16 to 21 years in a Chinese sample) [10, 22]. The mean age of CHR was 18.3 (SD = 1.5) years in this sample, which was similar to the sample of NAPLS [33], but younger than that of SHARP [10], and older than that of RAP [22]. The demographic characteristics of the participants enrolled in these studies may account for the age discrepancy. As the participants recruited in this study were college students with an age range of 14 to 31 years, while NAPLS recruited clinician-referred subjects aged 12 to 35 years, SHARP included help-seeking subjects aged 15 to 45 years, and RAP included help-seeking subjects aged 12 to 22 years.

Similar to previous studies using PQ-B to screen CHR in Chinese population [16, 30, 43–45], our research proved that PQ-B could be a feasible first-step tool to detect suspected CHR subjects in college students. Both the sensitivity and specificity of PQ-B were excellent in this study. Also, the results showed that 8 items of PQ-B including the PQ1 (asking “Do familiar surroundings sometimes seem strange, confusing, threatening or unreal to you?”), the PQ3 (asking “Do things that you see appear different from the way they usually do (brighter or duller, larger or smaller, or changed in some other way)?”), the PQ4 (asking “Have you had experiences with telepathy, psychic forces, or fortune telling?”), the PQ12 (asking “Do you worry at times that something may be wrong with your mind?”), the PQ13 (asking “Have you ever felt that you don’t exist, the world does not exist, or that you are dead?”), the PQ15 (asking “Do you hold beliefs that other people would find unusual or bizarre?”), the PQ17 (asking “Are your thoughts sometimes so strong that you can almost hear them?”) and the PQ18 (asking “Do you find yourself feeling mistrustful or suspicious of other people?”) had significant predictive value of SIPS diagnosis, which indicated that delusional ideas, perceptual abnormalities and suspiciousness were key symptoms in identifying CHR subjects. Similarly, Xu et al. [30] and Schultze et al. [27] also reported that perceptual abnormalities, delusional ideas and suspiciousness were the most prevalent psychosis-risk symptoms.

Nevertheless, our results recommended higher cutoff points for the total score and distress score of PQ-B (13 and 46) than those of SHARP did in the Chinese population (7 and 24, 30], which were also much higher than the cutoff points reported in the initial validation study of PQ-B (3 and 6, 16]. Despite the cultural background and language may lead to the differences of PQ-B cutoff points [46], there are still other reasons underlying the differences between the present study and SHARP. First, the participants in this study were college students who were tested with PQ-B in non-clinical settings (online or in the colleges), while SHARP recruited help-seeking outpatients from a psychological counselling center [30]. As prodromal symptoms mentioned in PQ-B were easily mixed with psychotic-like experiences (PLEs) that commonly existed in the general population [47–50], subjects who only had PLEs may also be tested positive with PQ-B, causing false positives. In addition, being rated in a clinical setting, rather than a non-clinical setting, may help the participants better understand the prodromal symptoms mentioned in the scale, and thus reducing false positive errors. Indeed, if we used the same cutoff points as SHARP did in their screening procedure, 72.0% (16,595/23063) of the participants would be screened positive with PQ-B in this study, while the positive rate of PQ-B in SHARP was 62.1% (1681/2705, 10], which may indicate that our false positive rate in PQ-B screening was higher. Second, the screening procedure differed. SHARP employed a 2-stage method where the PQ-B-positive subjects would all be invited to the SIPS interview, and 18.3% of the PQ-B-negative subjects were also interviewed [10]. However, we used a 3-stage method that applied a telephone interview to the PQ-B-positive subjects, and only subjects screened as positive in the telephone interview were invited to the SIPS interview. Moreover, no further investigations were provided to the negative subjects in the present study. These variance in the screening procedure may have contributed to the underestimation of CHR subjects, which would interfere the examination of the psychometric properties of PQ-B.

As discussed in the last paragraph, the telephone assessment can cause the missed diagnosis of CHR, which was a limitation of this study, whereas, it saved a lot of human resources in the process of CHR screening, in fact, particularly in a large sample of general population. It is worth mentioning that the telephone assessment employed in this study was not a semi-structured or structured interview that can be seen in other studies, although we also use SIPS as the assessing instrument [27, 51, 52]. In the present study, researchers only needed to ask questions following the SIPS subscale for positive symptoms, and to rate the severity for each domain based on the participants’ subjective reports. Consequently, it only took 5 min on average, which was much time-saving compared to a structured/semi-structured interview. In fact, it has also enhanced the screening efficiency by preventing almost 90% of the PQ-B-positive subjects from receiving a face-to-face SIPS interview, despite the fact that it was, of course, based on the sacrifice of the detection rate.

Besides, another limitation also lies in the screening process of GRDS, a subtype of the CHR syndromes. It’s worth noting that only participants who received the SIPS interview were screened for GRDS in this study. In other words, those participants who had family history of psychosis but did not manifest attenuate psychotic symptoms were neglected for the possible diagnosis of GRDS. Because for those first degree relative with psychosisor schizotypal personalitydisorder traits, a 30% drop in GAF score, rather than the attenuate psychosis symptoms is a necessity when diagnosing GRDS [12]. Therefore, the prevalence of CHR was underestimated in the present study.

Lastly, due to limited resources, the information received from the participants was incomplete, which is also a limitation of this study. On the one hand, we missed some important clinical features of the subjects, including family history and psychiatric comorbidity. On the other hand, the CHR subjects were not followed after being diagnosed, and therefore the transition to psychosis or other mental disorders was not investigated in this study.

## Conclusions

In conclusion, the 3-stage method is a useful strategy for screening CHR individuals in the general population, especially when the sample size is large. Among the college students, those with delusional ideas, perceptual abnormalities or suspiciousness were more likely to be screened as CHR subjects. However, several modifications should be made to the 3-stage method, especially for the screening of the familial high risk individuals, in order to enhance the sensitivity of the screening process, and further investigations comparing the 3-stage method with others can be done to explore more efficient ways of the screening. Also, for future researches, a longitudinal follow-up of the CHR subjects is essential to investigate the transition to psychosis of the CHR subjects, and to explore the related risk factors.

## Supplementary Information

**Additional file 1.**

## Data Availability

The dataset(s) generated during the current study are not publically available due to ethical restrictions but are available from the corresponding author on reasonable request.

## Abbreviations

CHR: Clinical high risk
UHR: Ultra-high risk
PQ-B: The prodromal questionnaire-brief version
SOPS: The scale of prodromal symptoms
SIPS: The structured interview for prodromal syndromes
CAARMS: The comprehensive assessment of at-risk mental states
SPI-A: The schizophrenia proneness instrument, adult
SPI-CY: The schizophrenia proneness instrument, child & youth version
PQ-16: The prodromal questionnaire-16 items (PQ-16)
EPS: The early psychosis screener
PLEs: Psychotic-like experiences
SMHC: Shanghai Mental Health Centre
TJH: Tongji Hospital of Tongji University
NBH: Nanjing Brain Hospital
FAHNU: The First Affiliated Hospital of Nanchang University
APSS: Attenuated positive symptom syndrome
BIPS: Brief Intermittent psychotic syndrome
GRDS: Genetic risk and deterioration syndrome
ROC: Receiver operating characteristic
AUCs: Areas under the curve
SHARP: The Shanghai-At-Risk-for-Psychosis Study
NAPLS: The North American Prodrome Longitudinal Study
RAP: The Recognition and prevention program

## References

[1] Sullivan HS (1927). The onset of schizophrenia. Am J Psychiatry 1994.

[2] McGlashan TH, Johannessen JO (1996). Early detection and intervention with schizophrenia: rationale. Schizophr Bull.

[3] Seidman LJ, Nordentoft M (2015). New Targets for Prevention of Schizophrenia: Is It Time for Interventions in the Premorbid Phase?. Schizophr Bull.

[4] Lieberman JA, Small SA, Girgis RR (2019). Early detection and preventive intervention in schizophrenia: from fantasy to reality. Am J Psychiatry.

[5] Perkins DO, Gu H, Boteva K, Lieberman JA (2005). Relationship between duration of untreated psychosis and outcome in first-episode schizophrenia: a critical review and meta-analysis. Am J Psychiatry.

[6] McGorry PD, Yung AR, Phillips LJ (2003). The “close-in” or ultra high-risk model: a safe and effective strategy for research and clinical intervention in prepsychotic mental disorder. Schizophr Bull.

[7] Cornblatt B, Lencz T, Obuchowski M (2002). The schizophrenia prodrome: treatment and high-risk perspectives. Schizophr Res.

[8] Fusar-Poli P (2017). The clinical high-risk state for psychosis (CHR-P), Version II. Schizophr Bull.

[9] Fusar-Poli P, Bonoldi I, Yung AR, Borgwardt S, Kempton MJ, Valmaggia L, Barale F, Caverzasi E, McGuire P (2012). Predicting psychosis: meta-analysis of transition outcomes in individuals at high clinical risk. Arch Gen Psychiatry.

[10] Zhang T, Li H, Woodberry KA, Seidman LJ, Zheng L, Li H, Zhao S, Tang Y, Guo Q, Lu X, Zhuo KM, Qian ZY, Chow A, Li CB, Jiang KD, Xiao ZP, Wang JJ (2014). Prodromal psychosis detection in a counseling center population in China: an epidemiological and clinical study. Schizophr Res.

[11] Schultze-Lutter F, Michel C, Schmidt SJ, Schimmelmann BG, Maric NP, Salokangas RK, Riecher-Rossler A, van der Gaag M, Nordentoft M, Raballo A (2015). EPA guidance on the early detection of clinical high risk states of psychoses. Eur Psychiatry.

[12] Miller TJ, McGlashan TH, Rosen JL, Cadenhead K, Cannon T, Ventura J, McFarlane W, Perkins DO, Pearlson GD, Woods SW (2003). Prodromal assessment with the structured interview for prodromal syndromes and the scale of prodromal symptoms: predictive validity, interrater reliability, and training to reliability. Schizophr Bull.

[13] Yung AR, Yuen HP, McGorry PD, Phillips LJ, Kelly D, Dell'Olio M, Francey SM, Cosgrave EM, Killackey E, Stanford C (2005). Mapping the onset of psychosis: the comprehensive assessment of at-risk mental states. Aust N Z J Psychiatry.

[14] Schultze-Lutter F, Ruhrmann S, tter: Development and evaluation of the schizophrenia proneness instrument, adult version (SPI-A). Schizophr Res 2006, 86(06):S4-S5, doi: 10.1016/S0920-9964(06)70014-7.

[15] Fux L, Walger P, Schimmelmann BG, Schultze-Lutter F (2013). The schizophrenia proneness instrument, child and youth version (SPI-CY): practicability and discriminative validity. Schizophr Res.

[16] Loewy RL, Pearson R, Vinogradov S, Bearden CE, Cannon TD (2011). Psychosis risk screening with the prodromal questionnaire--brief version (PQ-B). Schizophr Res.

[17] Ising HK, Veling W, Loewy RL, Rietveld MW, Rietdijk J, Dragt S, Klaassen RM, Nieman DH, Wunderink L, Linszen DH (2012). The validity of the 16-item version of the prodromal questionnaire (PQ-16) to screen for ultra high risk of developing psychosis in the general help-seeking population. Schizophr Bull.

[18] Miller T, Cicchetti D, Mcglashan T, Woods S (2004). Brief self-report screen to detect the schizophrenia prodrome. 12th Biennial Winter Workshop on Schizophrenia: 2004.

[19] Brodey BB, Addington J, First MB, Perkins DO, Woods SW, Walker EF, Walsh B, Nieri JM, Nunn MB, Putz J, Brodey IS (2018). The early psychosis screener (EPS): item development and qualitative validation. Schizophr Res.

[20] Brodey BB, Girgis RR, Favorov OV, Addington J, Perkins DO, Bearden CE, Woods SW, Walker EF, Cornblatt BA, Brucato G, Walsh B, Elkin KA, Brodey IS (2018). The early psychosis screener (EPS): quantitative validation against the SIPS using machine learning. Schizophr Res.

[21] Guo Y, Qu S, Qin H (2018). Study of the relationship between self-stigma and subjective quality of life for individuals with chronic schizophrenia in the community. Gen Psychiatr.

[22] Cornblatt BA, Carrion RE, Auther A, McLaughlin D, Olsen RH, John M, Correll CU (2015). Psychosis prevention: a modified clinical high risk perspective from the recognition and prevention (RAP) program. Am J Psychiatry.

[23] Zhang T, Xu L, Tang Y, Li H, Tang X, Cui H, Wei Y, Wang Y, Hu Q, Liu X, Li CB, Lu Z, McCarley RW, Seidman LJ, Wang JJ, on behalf of the SHARP (ShangHai At Risk for Psychosis) Study Group (2019). Prediction of psychosis in prodrome: development and validation of a simple, personalized risk calculator. Psychol Med.

[24] Zhang T, Li H, Tang Y, Li H, Zheng L, Guo Q, Zhao S, Zhuo K, Qian Z, Wang L, Dai YF, Chow A, Li CB, Jiang KD, Wang JJ, Xiao ZP (2015). Screening schizotypal personality disorder for detection of clinical high risk of psychosis in Chinese mental health services. Psychiatry Res.

[25] Chen F, Wang L, Heeramun-Aubeeluck A, Wang J, Shi J, Yuan J, Zhao X (2014). Identification and characterization of college students with attenuated psychosis syndrome in China. Psychiatry Res.

[26] Schultze-Lutter F, Michel C, Ruhrmann S, Schimmelmann BG (2014). Prevalence and clinical significance of DSM-5-attenuated psychosis syndrome in adolescents and young adults in the general population: the Bern epidemiological at-risk (BEAR) study. Schizophr Bull.

[27] Schultze-Lutter F, Michel C, Ruhrmann S, Schimmelmann BG (2018). Prevalence and clinical relevance of interview-assessed psychosis-risk symptoms in the young adult community. Psychol Med.

[28] Wang L, Shi J, Chen F, Yao Y, Zhan C, Yin X, Fang X, Wang H, Yuan J, Zhao X (2015). Family perception and 6-month symptomatic and functioning outcomes in young adolescents at clinical high risk for psychosis in a general population in China. Plos One.

[29] Loewy RL, Bearden CE, Johnson JK, Raine A, Cannon TD (2005). The prodromal questionnaire (PQ): preliminary validation of a self-report screening measure for prodromal and psychotic syndromes. Schizophr Res.

[30] Xu L, Zhang T, Zheng L, Li H, Tang Y, Luo X, Sheng J, Wang J (2016). Psychometric properties of prodromal questionnaire-brief version among Chinese help-seeking individuals. Plos One.

[31] Li-Na Z, Ji-Jun W, Tian-Hong Z, Hui L, Chun-Bo L, K-D JIANG (2012). Reliability and validity of the Chinese version of scale of psychosis-risk symptoms. Chin Ment Health J.

[32] Huang Y, Wang Y, Wang H, Liu Z, Yu X, Yan J, Yu Y, Kou C, Xu X, Lu J, Wang Z, He S, Xu Y, He Y, Li T, Guo W, Tian H, Xu G, Xu X, Ma Y, Wang L, Wang L, Yan Y, Wang B, Xiao S, Zhou L, Li L, Tan L, Zhang T, Ma C, Li Q, Ding H, Geng H, Jia F, Shi J, Wang S, Zhang N, du X, du X, Wu Y (2019). Prevalence of mental disorders in China: a cross-sectional epidemiological study. Lancet Psychiatry.

[33] Addington J, Cadenhead KS, Cannon TD, Cornblatt B, McGlashan TH, Perkins DO, Seidman LJ, Tsuang M, Walker EF, Woods SW (2007). North American Prodrome longitudinal study: a collaborative multisite approach to prodromal schizophrenia research. Schizophr Bull.

[34] Fusar-Poli P, Byrne M, Badger S, Valmaggia LR, McGuire PK (2013). Outreach and support in South London (OASIS), 2001-2011: ten years of early diagnosis and treatment for young individuals at high clinical risk for psychosis. Eur Psychiatry.

[35] Broome MR, Woolley JB, Johns LC, Valmaggia LR, Tabraham P, Gafoor R, Bramon E, McGuire PK (2005). Outreach and support in South London (OASIS): implementation of a clinical service for prodromal psychosis and the at risk mental state. Eur Psychiatry.

[36] Yung AR, Nelson B, Stanford C, Simmons MB, Cosgrave EM, Killackey E, Phillips LJ, Bechdolf A, Buckby J, McGorry PD (2008). Validation of “prodromal” criteria to detect individuals at ultra high risk of psychosis: 2 year follow-up. Schizophr Res.

[37] Gerstenberg M, Theodoridou A, Traber-Walker N, Franscini M, Wotruba D, Metzler S, Muller M, Dvorsky D, Correll CU, Walitza S (2016). Adolescents and adults at clinical high-risk for psychosis: age-related differences in attenuated positive symptoms syndrome prevalence and entanglement with basic symptoms. Psychol Med.

[38] Fusar-Poli P, De Micheli A, Cappucciati M, Rutigliano G, Davies C, Ramella-Cravaro V, Oliver D, Bonoldi I, Rocchetti M, Gavaghan L (2018). Diagnostic and prognostic significance of DSM-5 attenuated psychosis syndrome in Services for Individuals at ultra high risk for psychosis. Schizophr Bull.

[39] Spada G, Molteni S, Pistone C, Chiappedi M, McGuire P, Fusar-Poli P, Balottin U (2016). Identifying children and adolescents at ultra high risk of psychosis in Italian neuropsychiatry services: a feasibility study. Eur Child Adolesc Psychiatry.

[40] Salazar de Pablo G, Catalan A, Fusar-Poli P: Clinical Validity of DSM-5 Attenuated Psychosis Syndrome: Advances in Diagnosis, Prognosis, and Treatment. JAMA Psychiat. 2020;77(3):311–20. 10.1001/jamapsychiatry.2019.3561. 10.1001/jamapsychiatry.2019.3561 31746950

[41] Welham JL, Thomis RJ, McGrath JJ (2004). Age-at-first-registration for affective psychosis and schizophrenia. Schizophr Bull.

[42] Chan V (2017). Schizophrenia and psychosis: diagnosis, current research trends, and model treatment approaches with implications for transitional age youth. Child Adolesc Psychiatr Clin N Am.

[43] Fonseca-Pedrero E, Inchausti F, Perez-Albeniz A, Ortuno-Sierra J (2018). Validation of the prodromal questionnaire-brief in a representative sample of adolescents: internal structure, norms, reliability, and links with psychopathology. Int J Methods Psychiatr Res.

[44] Kline E, Thompson E, Demro C, Bussell K, Reeves G, Schiffman J (2015). Longitudinal validation of psychosis risk screening tools. Schizophr Res.

[45] Jang YE, Lee TY, Hur JW, Kwon JS (2019). Validation of the Korean version of the prodromal questionnaire-brief version in non-help-seeking individuals. Psychiatry Investig.

[46] Cicero DC, Krieg A, Martin EA (2019). Measurement invariance of the prodromal questionnaire-brief among white, Asian, Hispanic, and multiracial populations. Assessment.

[47] Fonseca-Pedrero E, Gooding DC, Ortuno-Sierra J, Paino M (2016). Assessing self-reported clinical high risk symptoms in community-derived adolescents: a psychometric evaluation of the prodromal questionnaire-brief. Compr Psychiatry.

[48] Shevlin M, Murphy J, Dorahy MJ, Adamson G (2007). The distribution of positive psychosis-like symptoms in the population: a latent class analysis of the National Comorbidity Survey. Schizophr Res.

[49] Rossler W, Riecher-Rossler A, Angst J, Murray R, Gamma A, Eich D, van Os J, Gross VA (2007). Psychotic experiences in the general population: a twenty-year prospective community study. Schizophr Res.

[50] Sullivan SA, Kounali D, Cannon M, David AS, Fletcher PC, Holmans P, Jones H, Jones PB, Linden DEJ, Lewis G, Owen MJ, O’Donovan M, Rammos A, Thompson A, Wolke D, Heron J, Zammit S (2020). A population-based cohort study examining the incidence and impact of psychotic experiences from childhood to adulthood, and prediction of psychotic disorder. Am J Psychiatry.

[51] Schultze-Lutter F, Schimmelmann BG, Fluckiger R, Michel C (2020). Effects of age and sex on clinical high-risk for psychosis in the community. World J Psychiatry.

[52] Michel C, Schmidt SJ, Schnyder N, Fluckiger R, Kaufeler I, Schimmelmann BG, Schultze-Lutter F (2019). Associations of psychosis-risk symptoms with quality of life and self-rated health in the community. Eur Psychiatry.

