# Supplementary material for: Screening of the college students at clinical high risk for psychosis in China: a multicenter epidemiological study
Source: BMC Psychiatry. 2021 May 17;21:253. doi: 10.1186/s12888-021-03229-8 (PMC8127262; doi:10.1186/s12888-021-03229-8)
Supplement: Supplementary file 1 — Additional file 1. [file 12888_2021_3229_MOESM1_ESM.docx]

**CHR-Screening Questionnaire**

**Demographic Information**

*Dear students, thanks for participating in this survey about personal mental health. Please read the instruction carefully and answer according to the actual situation. After this survey, we may conduct a telephone follow-up to further investigate your mental health state. Therefore, we hope that you leave the true and effective contact information so that we can get in contact with you.*

1. What’s your name/nickname?
2. Are you female or male?

□Female □Male

1. What’s your birthdate (yyyy/mm/dd)?
2. What’s your telephone number?
3. What’s your e-mail address?
4. Do you have a relative who is suffering (has suffered) from any type of psychiatric disorder?

□Yes □No

If YES, indicate who he/she is (the relationship between you and he/she) and which diagnosis it is/was?

**Prodromal Questionnaire-brief version (PQ-B)**

*Please indicate whether you have had the following thoughts, feelings and experiences in the past month by checking “yes” or “no” for each item. If you answer “YES” to an item, also indicate how distressing that experience has been for you.*

1. Do familiar surroundings sometimes seem strange, confusing, threatening or unreal to you?

□ YES □ NO

If YES: When this happens, I feel frightened, concerned, or it causes problems for me:

□ Strongly Disagree □ Disagree □ Neutral □ Agree □ Strongly Agree

1. Have you heard unusual sounds like banging, clicking, hissing, clapping or ringing in your ears?

□ YES □ NO

If YES: When this happens, I feel frightened, concerned, or it causes problems for me:

□ Strongly Disagree □ Disagree □ Neutral □ Agree □ Strongly Agree

1. Do things that you see appear different from the way they usually do (brighter or duller, larger or smaller, or changed in some other way)?

□ YES □ NO

If YES: When this happens, I feel frightened, concerned, or it causes problems for me:

□ Strongly Disagree □ Disagree □ Neutral □ Agree □ Strongly Agree

1. Have you had experiences with telepathy, psychic forces, or fortune telling?

□ YES □ NO

If YES: When this happens, I feel frightened, concerned, or it causes problems for me:

□ Strongly Disagree □ Disagree □ Neutral □ Agree □ Strongly Agree

1. Have you felt that you are not in control of your own ideas or thoughts?

□ YES □ NO

If YES: When this happens, I feel frightened, concerned, or it causes problems for me:

□ Strongly Disagree □ Disagree □ Neutral □ Agree □ Strongly Agree

1. Do you have difficulty getting your point across, because you ramble or go off the track a lot when you talk?

□ YES □ NO

If YES: When this happens, I feel frightened, concerned, or it causes problems for me:

□ Strongly Disagree □ Disagree □ Neutral □ Agree □ Strongly Agree

1. Do you have strong feelings or beliefs about being unusually gifted or talented in some way?

□ YES □ NO

If YES: When this happens, I feel frightened, concerned, or it causes problems for me:

□ Strongly Disagree □ Disagree □ Neutral □ Agree □ Strongly Agree

1. Do you feel that other people are watching you or talking about you?

□ YES □ NO

If YES: When this happens, I feel frightened, concerned, or it causes problems for me:

□ Strongly Disagree □ Disagree □ Neutral □ Agree □ Strongly Agree

1. Do you sometimes get strange feelings on or just beneath your skin, like bugs crawling?

□ YES □ NO

If YES: When this happens, I feel frightened, concerned, or it causes problems for me:

□ Strongly Disagree □ Disagree □ Neutral □ Agree □ Strongly Agree

1. Do you sometimes feel suddenly distracted by distant sounds that you are not normally aware of?

□ YES □ NO

If YES: When this happens, I feel frightened, concerned, or it causes problems for me:

□ Strongly Disagree □ Disagree □ Neutral □ Agree □ Strongly Agree

1. Have you had the sense that some person or force is around you, although you couldn’t see anyone?

□ YES □ NO

If YES: When this happens, I feel frightened, concerned, or it causes problems for me:

□ Strongly Disagree □Disagree □ Neutral □ Agree □ Strongly Agree

1. Do you worry at times that something may be wrong with your mind?

□ YES □ NO

If YES: When this happens, I feel frightened, concerned, or it causes problems for me:

□ Strongly Disagree □ Disagree □ Neutral □ Agree □ Strongly Agree

1. Have you ever felt that you don't exist, the world does not exist, or that you are dead?

□ YES □ NO

If YES: When this happens, I feel frightened, concerned, or it causes problems for me:

□ Strongly Disagree □ Disagree □ Neutral □ Agree □ Strongly Agree

1. Have you been confused at times whether something you experienced was real or imaginary?

□ YES □ NO

If YES: When this happens, I feel frightened, concerned, or it causes problems for me:

□ Strongly Disagree □ Disagree □ Neutral □ Agree □ Strongly Agree

1. Do you hold beliefs that other people would find unusual or bizarre?

□ YES □ NO

If YES: When this happens, I feel frightened, concerned, or it causes problems for me:

□ Strongly Disagree □ Disagree □ Neutral □ Agree □ Strongly Agree

1. Do you feel that parts of your body have changed in some way, or that parts of your body are working differently?

□ YES □ NO

If YES: When this happens, I feel frightened, concerned, or it causes problems for me:

□ Strongly Disagree □ Disagree □ Neutral □ Agree □ Strongly Agree

1. Are your thoughts sometimes so strong that you can almost hear them?

□ YES □ NO

If YES: When this happens, I feel frightened, concerned, or it causes problems for me:

□ Strongly Disagree □ Disagree □ Neutral □ Agree □ Strongly Agree

1. Do you find yourself feeling mistrustful or suspicious of other people?

□ YES □ NO

If YES: When this happens, I feel frightened, concerned, or it causes problems for me:

□ Strongly Disagree □ Disagree □ Neutral □ Agree □ Strongly Agree

1. Have you seen unusual things like flashes, flames, blinding light, or geometric figures?

□ YES □ NO

If YES: When this happens, I feel frightened, concerned, or it causes problems for me:

□ Strongly Disagree □ Disagree □ Neutral □ Agree □ Strongly Agree

1. Have you seen things that other people can't see or don't seem to see?

□ YES □ NO

If YES: When this happens, I feel frightened, concerned, or it causes problems for me:

□ Strongly Disagree □ Disagree □ Neutral □ Agree □ Strongly Agree

1. Do people sometimes find it hard to understand what you are saying?

□ YES □ NO

If YES: When this happens, I feel frightened, concerned, or it causes problems for me:

□ Strongly Disagree □ Disagree □ Neutral □ Agree □ Strongly Agree
